# Immunomodulation of MSCs and MSC-Derived Extracellular Vesicles in Osteoarthritis

**DOI:** 10.3389/fbioe.2020.575057

**Published:** 2020-10-29

**Authors:** Xige Zhao, Yanhong Zhao, Xun Sun, Yi Xing, Xing Wang, Qiang Yang

**Affiliations:** ^1^Stomatological Hospital of Tianjin Medical University, Tianjin, China; ^2^Department of Spine Surgery, Tianjin Hospital, Tianjin University, Tianjin, China; ^3^Beijing National Laboratory for Molecular Sciences, Institute of Chemistry, Chinese Academy of Sciences, Beijing, China; ^4^University of Chinese Academy of Sciences, Beijing, China

**Keywords:** osteoarthritis, MSCs, exosomes, immunomodulatory, macrophage

## Abstract

Osteoarthritis (OA) has become recognized as a low-grade inflammatory state. Inflammatory infiltration of the synovium by macrophages, T cells, B cells, and other immune cells is often observed in OA patients and plays a key role in the pathogenesis of OA. Hence, orchestrating the local inflammatory microenvironment and tissue regeneration microenvironment is important for the treatment of OA. Mesenchymal stem cells (MSCs) offer the potential for cartilage regeneration owing to their effective immunomodulatory properties and anti-inflammatory abilities. The paracrine effect, mediated by MSC-derived extracellular vehicles (EVs), has recently been suggested as a mechanism for their therapeutic properties. In this review, we summarize the interactions between MSCs or MSC-derived EVs and OA-related immune cells and discuss their therapeutic effects in OA. Additionally, we discuss the potential of MSC-derived EVs as a novel cell-free therapy approach for the clinical treatment of OA.

## Introduction

Osteoarthritis (OA) is a chronic degenerative joint disease that affects about 15% of the global population (Loeser, 2013; Hunter and Bierma-Zeinstra, 2019). OA involves not only the knees but also the spine, hands, and temporal–mandibular joints and is characterized by the articular cartilage degradation, synovitis, subchondral bone remodeling, and osteophyte formation. Patients experience aggravating pain and disability, resulting in decreased life quality and a high economic burden. The traditional viewpoint is that OA is a non-inflammatory disease. However, studies have shown that there is a type of chronic, low-grade inflammation in the pathogenesis of OA, which seriously hinders the proliferation of chondrocytes and the deposition of cartilage matrix, resulting in low efficiency of repair (Aspden and Saunders, 2019; Chow and Chin, 2020; Woodell-May and Sommerfeld, 2020). Various immune cells have been identified in the synovium of OA patients. Among them, macrophages, T cells, and B cells are the most abundant (de Lange-Brokaar et al., 2012).

Synovial macrophages (Mφ) are the main immune cells in joints (Fernandes et al., 2020; Wu C. L. et al., 2020). Historically, macrophages have been characterized as classically activated macrophages, or proinflammatory macrophages (M1Mφ), and are activated by interferon-γ (IFN-γ), tissue necrosis factor-α (TNF-α), or pathogen-associated molecular patterns. Once activated, these macrophages secrete proinflammatory cytokines such as interleukin-1 (IL-1), IL-6, IL-12, and inducible nitric oxide synthase. Conversely, anti-inflammatory macrophages (M2Mφ) are activated by an alternative activation pathway. These macrophages release growth and angiogenic factors such as transforming growth factor-β (TGF-β) and arginase-1 (Arg-1), which down-regulate inflammation and promote remodeling of damaged tissue (Wynn and Vannella, 2016; Oishi and Manabe, 2018). The spatiotemporal distribution of M1 and M2Mφ plays a key role in the orchestration of inflammation and tissue regeneration (Zhang H. Y. et al., 2018; Fernandes et al., 2020). It has been thought that depletion of Mφ can eliminate inflammation and further alleviate the progression of OA. For this reason, Wu et al. used the macrophage Fas-induced apoptosis (MaFIA) transgenic mice. They placed the MaFIA mice on a high-fat diet and induced knee OA and then conditional macrophage exhaustion in synovium tissue. The results showed that OA with obesity was not attenuated by macrophage depletion of both M1 and M2 types. On the contrary, inflammatory cytokine production was intensified, which induced an increase of joint synovitis and cartilage damage (Wu et al., 2017). Given that M1Mφ is involved mainly in the initiation of inflammation, yet M2Mφ is involved mainly in the regression of inflammation, this phenomenon indicated that the failure of transformation from M1Mφ to M2Mφ may result in persistent chronic inflammation. At present, the implications of the alteration of the Mφ polarization state for OA treatment are not clear, but several relevant studies have shown that infiltrative M1Mφ and M2Mφ exert different effects on cells within the joint, thus playing a key role in the orchestration of inflammation and regeneration (Blom et al., 2007; Fahy et al., 2014; Fichadiya et al., 2016; Utomo et al., 2016; Samavedi et al., 2017; Hu et al., 2018; Chen et al., 2020).

**Figure 1:**
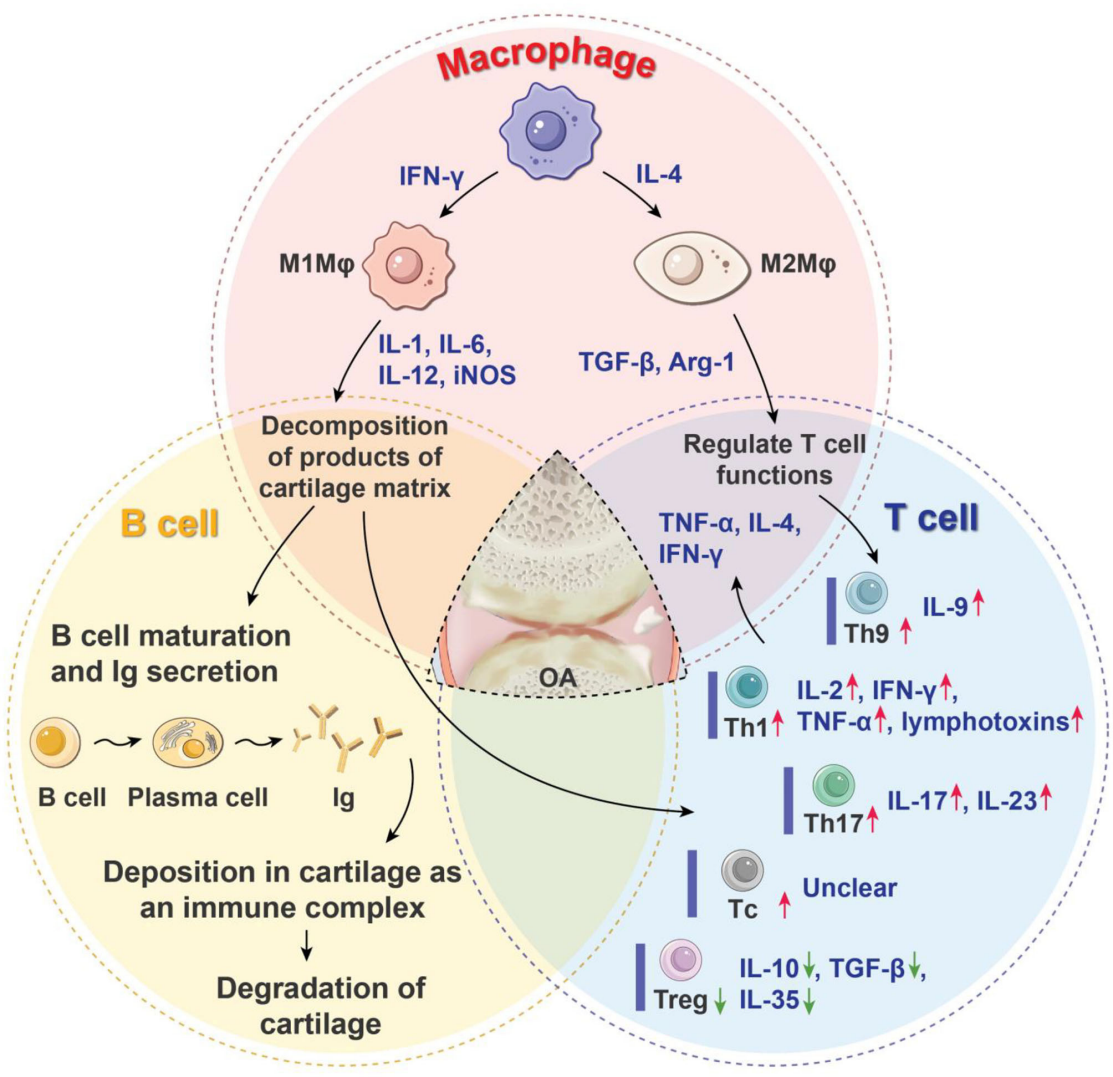
Roles of immune cells in the progression of OA. OA is characterized by the articular cartilage degeneration, synovitis, subchondral bone sclerosis, and osteophyte formation. Macrophages, T cells, and B cells are the three main immune cells that regulate inflammatory processes and the immune response of OA. They form a complex network through coordination or antagonism. Arg-1, arginase-1; IL, interleukin; Ig, immune globulin; IFN-γ, interferon-γ; iNOS, inducible nitric oxide synthase; Mφ, macrophages; OA, osteoarthritis; Tc, cytotoxic T cell; T_H_, helper T cell; Treg, regulatory T cell; TGF-β, transforming growth factor-β; TNF-α, tissue necrosis factor-α; red arrow means increased production of proinflammatory cells or mediators; green arrow means decreased production of anti-inflammatory cells or mediators.

T cells are lymphocytes that represent the second main constituents of synovial infiltrates in OA patients. T cells can be broadly divided into helper T cells (T_H_ cells), cytotoxic T cells (Tc cells), and regulatory T cells (Tregs) according to their different functions, and the first two are collectively known as effector T cells. Previous studies have shown that both CD4^+^ (mainly differentiated into T_H_ cells after activation) and CD8^+^ T-cell subsets (mainly differentiated into Tc cells after activation) have been found at higher levels not only in synovial fluid and membranes but also in peripheral blood (Haynes et al., 2002; Hussein et al., 2008; Labinsky et al., 2020), which suggests that T_H_ cells and Tc cells are involved in the pathogenesis of OA (Raphael et al., 2015). Specifically, OA synovial tissue exhibits increased levels of T_H_1 (CD4^+^IFN-γ^+^ T cells), T_H_17 (CD4^+^IL-17^+^ T cells), and Tc (CD8^+^ T cells), whereas the synovial fluid has increased levels of T_H_1, T_H_9 (CD4^+^IL9^+^ T cells) and T_H_17 cells. T_H_1 produce IL-2, IFN-γ, TNF-α, lymphotoxins, and granulocyte–macrophage colony-stimulating factor Inflammatory cytokines, which exacerbate joint inflammation and recruit more effector T cells to inflammatory tissues, thus causing tissue damage (Li et al., 2017). T_H_9 cells preferentially produce IL-9, which is positively correlated with OA index (Qi et al., 2016). T_H_17 cells mainly produce IL-17 and IL-23, and the overexpression of the two cytokines promotes the infiltration of the immune cells, secretion of vascular endothelial growth factor, and growth of the blood vessel, thus aggravating cartilage degeneration (Bommireddy and Doetschman, 2007). Tc cells also have been found to significantly shape the pathogenesis of OA; however, the exact role of them response in the biology of OA is still unclear (Hsieh et al., 2013). Under the induction of TGF-β, naive T cells differentiate into Tregs. Tregs are important immunomodulators in many inflammatory and autoimmune diseases, as they inhibit the immune response and maintain immune tolerance by secreting cytokines such as TGF-β, IL-10, and IL-35. Tregs exhibit a decreased response in the pathogenesis of OA (Hori et al., 2003; Bommireddy and Doetschman, 2007). Besides, the role of T_H_17/Treg ratio in the immunoregulation of OA has also attracted increasing attention. It was found that if T_H_17/Treg ratio is deviated in favor of proinflammatory T_H_17 cells, arthritis was exacerbated (Noack and Miossec, 2014; Yang et al., 2018; Min et al., 2019; Peter et al., 2020) (Figure 1).

In the case of OA, if the decomposition products of the cartilage matrix are exposed, B cells can be stimulated to produce autoantibodies and activate humoral immunity (Zhu et al., 2020), leading to disturbance of the entire articular environment. In such cases, autoantibodies against cartilage mesothelin, osteopontin, ykl-39, collagen, and proteoglycan have been detected (Ozeki et al., 2016). The harmful effects of autoantibody formation may lie in its diffusion and deposition in articular cartilage as an immune complex, which makes it easy to degrade and destroy. This may stimulate the complement system to clear the complex and become part of the complex pathogenic circuit that causes tissue damage (Modinger et al., 2019) (Figure 1).

Therefore, the treatment of OA should focus not only on “cartilage wear” but also on the immune cells that have a major impact on the occurrence and development of OA. Regulating the local inflammatory microenvironment and tissue regeneration microenvironment is important in the treatment of OA. Mesenchymal stem cells (MSCs) with self-renewal capacity and multidifferentiation potential have received much attention as an alternative approach in the management of cartilage degradation (Barry and Murphy, 2013; Fernandez-Pernas et al., 2020). Moreover, MSCs have been shown to possess effective immunomodulatory properties in the treatment of inflammatory diseases (Guillamat-Prats et al., 2017; de Castro et al., 2019; Zhao L. et al., 2019) and are capable of regulating the immune cells that play a pathogenic role in the progression of OA. In recent years, the paracrine effect of MSCs mediated by extracellular vehicles (EVs) has been suggested to explain their curative effect (Kalluri and LeBleu, 2020). Three main types of EVs have been described based on their biogenesis and size: exosomes (30–150 nm in diameter), microvesicles/microparticles, and apoptotic bodies (both considered to be >100 nm). Exosomes are secreted to the extracellular environment through the fusion of multivesicular bodies with the plasma membrane. The last two types of vesicles are released through forward budding of the plasma membrane in living and dying cells, respectively (Bui et al., 2018; Kalluri and LeBleu, 2020). EVs, as a natural and efficient transport carrier, can maintain functional characteristics similar to their parent cells (Bui et al., 2018; Wu X. et al., 2020). This insight has given rise to a new paradigm wherein EVs are collected from MSCs and used to actively regulate complicated communication among components of the immune system (Wang et al., 2018). The cell-free nature of EVs suggests that they may have a more favorable safety profile than cell-based therapies.

Within this review, we summarize and discuss what is known about the interactions of MSCs or MSC-derived EVs with OA-related immune cells and the importance of these interactions in therapeutic strategies for OA.

## Roles of MSCs in Immunomodulation

MSCs have immunomodulatory properties relying on both cell-to-cell contact and paracrine signaling. Furthermore, under the local inflammatory microenvironment, low or high levels of specific toll-like receptor (TLR) ligands may greatly influence the immunoregulatory characteristics of MSCs and thereby impact the effect of MSC-based therapies (Lei et al., 2011). In the early stage of OA, the molecules produced by damaged tissue or pathogens will be recognized by TLR2/4 (differences by species may exist) on the surface of MSCs, lead to MSCs polarization into MSC1 (Tomchuck et al., 2008). This phenotype can promote the immune response in the early stage of inflammation. This kind of acute inflammatory response after injury can induce the accumulation of inflammatory cells in the damaged area and play a role of automatic debridement, which has characteristics of protecting the joint tissue. However, persistent chronic inflammation is harmful to joints (Huang and Kraus, 2016). Accordingly, in the late stage of inflammation, the high levels of TLR3 ligand lead to MSCs transform to the inflammation-dampening MSC2 phenotype to prevent prolonged damage to joints (Ayala-Cuellar et al., 2019). Failure of transformation in the stage of inflammation abatement during OA is the key to the continuation of the pathological vicious circle. In this case, joint resident MSCs may not be as effective as infused MSCs in restoring immunological and regenerative homeostasis in the degenerative joint. Therefore, in this section, we summarize the major studies investigating the effects of adoptive transferred MSCs during OA or other chronic degenerative diseases with chronic inflammation, focusing mainly on their immunosuppressive properties.

### Immunomodulatory Effect of MSCs on Macrophages

MSCs can produce various growth factors, chemokines, and other signaling molecules affecting macrophage polarization, maturation, proliferation, and migration, which have been verified by MSC secretome analyses (Vizoso et al., 2017; Lin and Du, 2018).

Recent studies have revealed the ability of MSCs to regulate macrophage polarization by inducing a transformation from the M1 to the M2 phenotype, thus promoting the healing process (Cho et al., 2014). Abumaree et al. co-cultured “fried egg–like” M1 macrophages with MSCs for 3 days, and the cells gradually turned into “spindle-like” M2 macrophages (Abumaree et al., 2013). This process is accompanied by high-level secretion of IL-10, low levels of IL-12 and IL-1β, and increased phagocytic activity. MSCs can also regulate the immune phenotype of macrophages *in vivo*, which has been demonstrated in a variety of disease models. For example, MSCs can prolong corneal allograft survival by altering M1/M2 polarization and inhibiting the infiltration of antigen-presenting cells (APCs) (Murphy et al., 2016).

**Figure 2:**
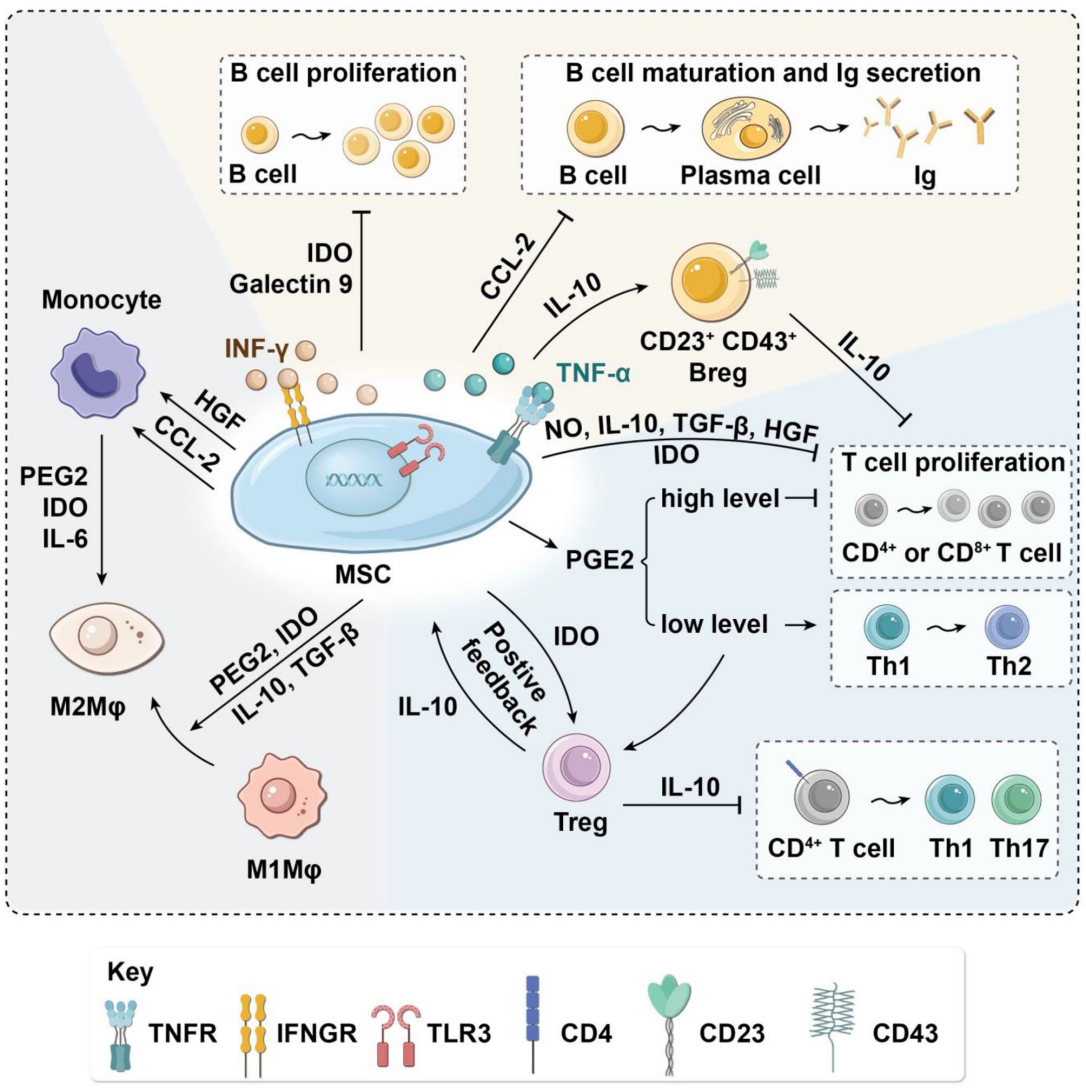
Immunoregulatory effects of MSCs on immune cells in the paracrine manner. MSCs have immunomodulatory effects on OA-related macrophages, T cells, and B cells via the secretion of various cytokines. MSCs suppress macrophage migration and regulate their polarization by inducing transformation from M1 to M2. MSCs inhibit the proliferation of T cells and activation of inflammatory T_H_1 and T_H_17 cells. In turn, MSCs induce immunosuppressive Tregs. Moreover, MSCs decrease B-cell activation and proliferation and attenuate immunoglobulin production. Arg-1, arginase-1; Breg, regulatory B cell; CCL-2, C-C motif chemokine ligand 2; HGF, hepatocyte growth factor; IDO, indoleamine-2,3-dioxygenase; IL, interleukin; Ig, immune globulin; IFN-γ, interferon-γ; iNOS, inducible nitric oxide synthase; MSC, mesenchymal stem cell; Mφ, macrophages; NO, nitric oxide; OA, osteoarthritis; PGE2, prostaglandin E2; T_H_, helper T cell; Treg, regulatory T cell; TGF-β, transforming growth factor-β.

At present, the mechanism by which MSCs regulate the phenotypic transformation of macrophages is not fully understood. Several studies have confirmed that MSCs affect macrophage polarization via direct cell contact and cytokines such as IL-10, prostaglandin E2 (PGE2), indoleamine 2,3-dioxygenase (IDO), and TGF-β secreted by MSCs (Park et al., 2018; Shi et al., 2019). IL-10 can promote the inflammatory repair of tissues, reduce inflammatory damage, and promote the transformation of macrophages to M2, which may be one of the main mechanisms of MSC-mediated macrophage transformation (Faulknor et al., 2017). PGE2 is another important anti-inflammatory factor; it can combine with EP2 and EP4 receptors on the surface of macrophages, change the expression of downstream genes, and cause macrophages to transform into the M2 phenotype (Kawahara et al., 2015). The anti-inflammatory effect of macrophages is weakened by using specific PGE2 inhibitors, and the expression of IL-10 and IL-1α in anti-inflammatory cells also significantly decreases (Ylostalo et al., 2012). MSCs can also release IDO, which induces macrophages to transform into the M2 phenotype and release more IL-10. Lee et al. found that after treatment with the IDO-specific inhibitor 1-l-methyl-tryptophan (1-l-MT), the expression of IFN-α increased, and anti-inflammatory effects were significantly inhibited (Lee et al., 2016). TGF-β1 is an important marker for M2 monocytes/macrophages. Liu et al. found that the inflammation was significantly attenuated after TGF-β1 injection in colitis model mice (Jiang and Xu, 2020). Moreover, MSCs have also been reported to induce monocyte maturation directly to M2 macrophages by MSC secretion of IDO, IL-6, and PGE2 (Figure 2).

Collectively, these studies show that MSCs can induce macrophages to transform from the M1 to the M2 phenotype, inhibit proinflammatory cytokines, release anti-inflammatory cytokines, reduce joint tissue damage, suppress the progress of inflammation, and promote the repair of inflammatory tissue.

### Immunomodulatory Effects of MSCs on T Cells

#### MSCs Inhibit T-Cell Proliferation

Nitric oxide (NO) secretion by mouse MSCs has been shown to regulate immunosuppression of T-cell responses directly by cell cycle arrest or apoptosis (Sato et al., 2007). Cytokines such as TGF-β and hepatocyte growth factor secreted by MSCs can down-regulate the expression of cyclin D2 and up-regulate the expression of p27kip1 in T cells, resulting in cell cycle arrest in the G1 phase, thus inhibiting T-cell proliferation (Glennie et al., 2005; Kyurkchiev et al., 2014). PGE2 secreted by MSCs can also inhibit T-cell proliferation by decreasing IL-2 and down-regulating IL-2 receptor, leading to impaired DNA binding activity of transcription factors through inhibition of Janus kinase 3 signaling (Burr et al., 2013). Under stimulation by proinflammatory factors such as TNF-α and IFN-γ, MSCs can inhibit the proliferation of T cells and induce the apoptosis of activated T cells by producing IDO (Behm et al., 2020), an iron-containing heme monomer membrane-binding protein that can catalyze the transformation of tryptophan into l-canine uric acid and picolinic acid. Tryptophan is an essential amino acid for the maintenance of T-cell activation and proliferation (Liu et al., 2016). The proliferation of T cells is blocked in the G1 phase if tryptophan concentration is low, resulting in the decrease of T cells. The tryptophan depletion mediated by IDO inhibits only activated T cells, not resting T cells (Figure 2).

#### MSCs Modulate T Cell Activation, Differentiation, and Effector Function

Cytokines secreted by MSCs can not only inhibit proliferation and induce apoptosis of T cells but also suppress the activation of naive T cells and change the differentiation process of T-cell subsets. Specifically, MSC-secreted cytokines may inhibit proinflammatory T cells and induce Tregs (Kalinski, 2012; Luz-Crawford et al., 2013; Baharlou et al., 2019; Jimenez-Puerta et al., 2020) resulting in decreased production of TNF-α and IL-12 and increased production of IL-10. When the concentration of IL-10 in the microenvironment reaches a certain level, soluble human leukocyte antigen-G5 (HLA-G5) will be secreted, attenuating the activation of CD4^+^ T cells to T_H_1 and T_H_17 and inducing Treg production (Selmani et al., 2008), thus forming a positive feedback loop (Figure 2).

**Figure 3:**
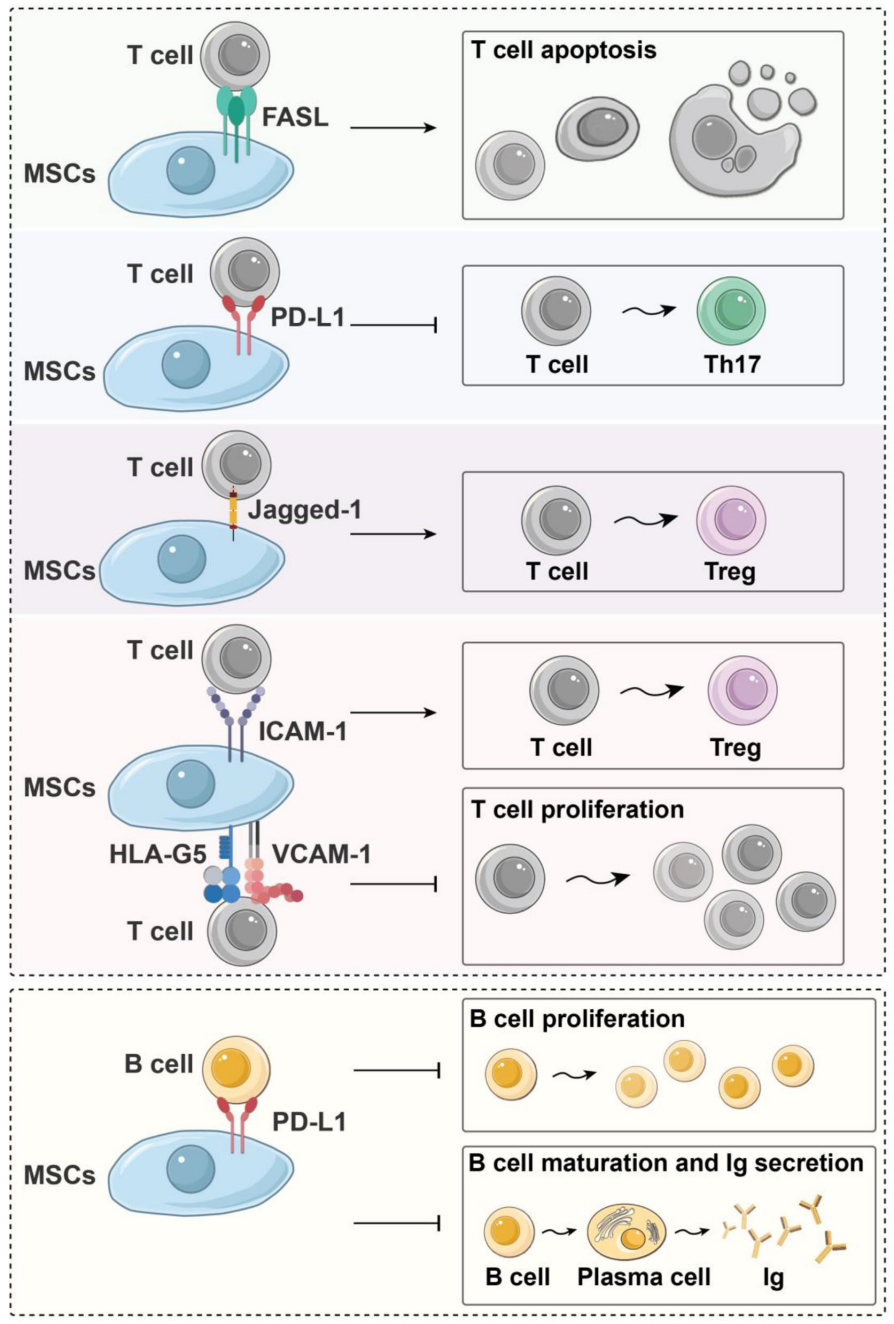
Immunoregulatory effects of MSCs on immune cells in a contact-dependent manner. Fas/FasL signal activation can induce the apoptosis of inflammatory T cells; Jagged-1/Notch-1 signal activation can induce CD4^+^ T cells to differentiate into Tregs, and PD-1/PD-L1 signaling effectively represses T_H_17 differentiation and inhibits the proliferation and maturation of B cells. Moreover, HLA-G5, ICAM-1, and VCAM-1 are needed for MSCs to exert their immunosuppressive effects upon T cells. HLA-G5, human leukocyte antigen-G5; ICAM-1, intercellular adhesion molecule-1; Ig, immune globulin; MSC, mesenchymal stem cells; PD-L1, programmed death-1 ligand; T_H_, helper T cell; Treg, regulatory T cell; VCAM-1, vascular cell adhesion molecule-1.

Direct contact between MSCs and T cells also plays an important role in the immune regulation of T cells. The expression of intercellular adhesion molecule-1 and vascular cell adhesion molecule-1 (VCAM-1) is indispensable for MSCs to exert immunosuppressive effects toward T cells via increasing the adhesion between MSCs and T cells (Ren et al., 2010; Ma et al., 2017). In addition, some cell-to-cell contact-dependent mechanisms participate in the immunomodulation of T cells: Fas/FasL signaling pathway activation can induce the apoptosis of inflammatory T cells (Akiyama et al., 2012), Jagged-1/Notch-1 signal activation can promote CD4^+^ T-cell differentiation into Tregs (Del Papa et al., 2013; Cahill et al., 2015), and programmed death-1/programmed death-1 ligand (PD-1/PD-L1) signaling effectively represses T_H_17 differentiation (Kim et al., 2018). Moreover, direct contact between MSCs and T cells requires HLA-G5, which is responsible for both the contact-dependent and paracrine immunosuppressive functions of MSCs (Figure 3) (Selmani et al., 2008).

In summary, MSCs inhibit the proliferation of T cells and the activation of inflammatory T_H_1 and T_H_17 cells. In turn, MSCs induce immunosuppressive Tregs, thus inhibiting the immune response and attenuating inflammation.

### Immunomodulatory Effects of MSCs on B Cells

Both secreted factors and cell-to-cell contact are needed for MSC modulation of B cells. Che et al. showed that co-culture with MSCs inhibits the phosphorylation of serine/threonine kinase and p38 mitogen-activated protein kinase in B cells (Che et al., 2012), while promoting the phosphorylation of extracellular response kinase 1/2 (ERK1/2) (Tabera et al., 2008). Another recent study showed that co-culture with MSCs significantly enhances the immunomodulatory activity of B cells by up-regulating IL-10. Chen et al. found that MSCs can induce a new regulatory B-cell (Breg) population characterized by CD23^+^CD43^+^ phenotypes. These CD23^+^CD43^+^ Breg cells significantly inhibit the secretion of proinflammatory cytokines and the proliferation of T cells through the IL-10–dependent pathway (Carreras-Planella et al., 2019a; Chen et al., 2019). Rafei et al. found that MSCs inhibit the expression of transcription factor signal transducer and activator of transcription 3 (STAT3) via chemokine C-C motif chemokine ligand 2 (CCL2) and then induce paired box 5 (PAX5) protein synthesis to inhibit the secretion of immune globulin in plasma cells (Rafei et al., 2008) (Figure 2). In addition to the paracrine effect, MSCs also play a contact-dependent immunomodulatory role with B cells. Schena et al. found that when activated by a high concentration of IFN-γ, MSCs can activate the programmed cell death receptor through direct contact and that the PD-1/PD-L1 signaling pathway inhibits the proliferation and maturation of B cells (Figure 3) (Schena et al., 2010).

In conclusion, MSCs can inhibit the proliferation of B cells and the differentiation and maturation of plasma cells and the secretion of antibody, while inducing a new Breg population, and attenuate inflammation in osteoarthritic joints.

## MSC-Derived EVS as Immunomodulatory Therapeutics

### Immunomodulatory Effects of MSC-Derived EVs on Macrophages

Several studies have shown that MSC-derived EVs can blunt the recruitment of macrophages. For instance, Shen et al. found that MSC-derived EVs express C-C motif chemokine receptor 2, which plays a key role in preventing macrophage accumulation and tissue damage by binding and serving as a decoy to inhibit proinflammatory chemokine CCL2 activity (Shen et al., 2016).

In addition, the therapeutic efficacy of MSC-derived EVs targeting M1/M2 polarization has been studied in various disease models. Zhang et al. found that EVs derived from MSCs have an immunomodulatory effect and can attract M2 macrophages to infiltrate OA cartilage defects and synovium, reduce the infiltration of M1 macrophages, and down-regulate the expression of IL-1β and TNF-α, thereby inhibiting the inflammatory response in OA (Zhang S. et al., 2018). In spinal cord injury (SCI) models, the detection of fluorescence-labeled human umbilical cord MSC (hUC-MSC)–derived EVs after intravenous infusion showed that hUC-MSC–derived EVs migrate to the injury site in a manner similar to hUC-MSCs themselves. Macrophages, especially the M2 subtype, are the primary target of EVs at SCI sites (Lankford et al., 2018). Sun et al. further confirmed that hUC-MSC–derived EVs can induce macrophage polarization from M1 to M2 and down-regulate the release of inflammatory factors, facilitating the repair of SCI (Sun et al., 2018). In an experimental murine model of bronchopulmonary dysplasia, MSC-derived EVs ameliorated hyperoxia-associated inflammation via modulation of lung macrophage phenotype (Willis et al., 2018). Similar immunoregulatory roles of MSC-derived EVs have been established in inflammatory bowel disease (Schena et al., 2010) and diabetic peripheral neuropathy (Fan et al., 2020).

Several studies have further explored the immunoregulatory mechanism in MSC-derived EVs. Zhao et al. found that mouse bone marrow MSC-derived EVs attenuate myocardial ischemia/reperfusion (I/R) injury in mice via shuttling microRNA-182 (miR-182), which targeted TLR4/nuclear factor κB/PI3K/Akt signaling, thereby modifying the polarization status of macrophages (Zhao J. et al., 2019). Another study demonstrated that adipose-derived stem cell (ADSC)–derived EVs facilitate the transportation of EV-enriched active STAT3 into macrophages to regulate polarization to the M2 phenotype through the transactivation of Arg-1 (Zhao et al., 2018). A recent study on cutaneous wound healing showed that miR-223, which is contained in MSC-derived EVs, induces the polarization of macrophages by targeting pknox1 (He et al., 2019).

Preconditioning MSCs with stimulating factors may increase the therapeutic potential of EVs. Ti et al. found that MSC-derived EVs preconditioned with lipopolysaccharide (LPS-EVs) had a more significant regulatory effect on macrophage polarization. The results of microarray analysis showed that the LPS-EVs mechanism of immunomodulation is associated with the expression of let-7b, which can impede the TLR4 pathway to regulate macrophage polarization (Ti et al., 2015). Song et al. used IL-1β to stimulate hUC-MSCs before administration to septic mice and found that huMSCs transfer miR-146a to macrophages via EVs, thereby promoting the M1-M2 transition. The survival rate of mice in the EV-treated group was decreased after miR-146a inhibition, demonstrating that miR-146a plays a protective role in the treatment of sepsis by MSC-derived EVs (Song et al., 2017). Transfection is another method of MSC modification. Jiang et al. found that miR-30d-5p–overexpressing ADSC-derived EVs prevent cerebral injury by inhibiting autophagy-mediated microglial polarization to M1 via the inhibition of Beclin-1 and autophagy-related gene 5 (ATG5) (Jiang et al., 2018).

These studies indicate that MSC-derived EVs can regulate macrophage polarization in a manner similar to MSCs. Moreover, considering that MSCs have plasticity, the EVs from preconditioned MSCs may possess different characteristics and may obtain better therapeutic effects (Table 1).

**Table 1:** Effects of MSC-derived EVs on OA-related immune cells.

**Disease model**	**Extracellular vesicle source**	**Precondition of MSCs**	**Effects on immune cells**	**Defined key factors**	**References**
Renal injury	Mouse BMSC	\	Suppress the recruitment and action of macrophages	CCR2	Shen et al., 2016
Osteochondral defects	Human ESC-MSC	\	Induce the infiltration of M2 Mφ and reduce the infiltration of M1 Mφ in the defects	\	Zhang B. et al., 2018
Spinal cord injury	Human UC-MSC	\	Induce Mφ polarization from M1 to M2 and down-regulate the release of inflammatory factors	\	Sun et al., 2018
Experimental bronchopulmonary dysplasia	Mouse BMSC	\	Decrease and increase M1 and M2 Mφ phenotype markers, respectively	\	Willis et al., 2018
IBD	Human BMSC	\	Metallothionein-2 acts as a critical negative regulator of the inflammatory response in Mφs.	Metallothionein-2	Liu et al., 2019
DPN	Mouse BMSC	\	Decrease and increase M1 and M2 Mφ phenotype markers, respectively	miR-17, miR-23a, miR-125b	Fan et al., 2020
Myocardial I/R injury	Mouse BMSC	\	Mediate macrophage polarization from M1 to M2	miR-182	Zhao J. et al., 2019
Obesity-induced inflammation	Mouse ADSC	\	Induce M2 Mφ polarization	Activated STAT3	Zhao et al., 2018
Skin defect	Human jaw BMSC	\	Induce M2 Mφ polarization	miR-223	He et al., 2019
Diabetic cutaneous wounds	Human UC-MSC	Stimulated by LPS	Induce M2 Mφ polarization	let-7b	Ti et al., 2015
Sepsis	Human UC-MSC	Stimulated by IL-1β	Induce M2 Mφ polarization	miR-146a	Song et al., 2017
Middle cerebral artery occlusion	Rat ADSC	Transfection of miR-30d-5p mimic	Transform microglial/macrophage polarization from M1 to M2	miR-30d-5p	Jiang et al., 2018
\	Human BMSC	\	Induce the transformation of T_H_1 cells into T_H_2 cells, reduce the potential of T cells to differentiate into T_H_17 cells and increase the content of Tregs	\	Chen et al., 2016
Arthritis (DTH or CIA induced)	Mouse BMSC	\	Inhibit T-cell proliferation through Treg induction. Suppress plasma cell differentiation and induce Bregs	\	Cosenza et al., 2018
GVHD	Human ESC-MSC	\	Induce the differentiation of naive T cells into Tregs	\	Zhang B. et al., 2018
EAE	Human BMSC	Stimulated by IFN-γ	Suppress T Cell Proliferation and up-regulate the number of Tregs within the spinal	Aggrecan, periostin, HAPLN1	Riazifar et al., 2019
Myocardial I/R injury	Human UC-MSC	Transfection of miR-181 mimic	Induce the differentiation of Tregs	miR-181	Wei et al., 2019
\	Human BMSC	\	Inhibit the proliferation of B cells and decrease the chemotaxis of B cells	CXCL8, MZB1	Khare et al., 2018

*ADSC, adipose-derived stem cells; BMSC, bone marrow mesenchymal cells; Breg, regulatory B cell; CCR2, C-C chemokine receptor type 2; CIA, collagen-induced arthritis; CXCL8, C-X-C motif ligand 8; DTH, delayed-type hypersensitivity; EAE, experimental autoimmune encephalomyelitis; ESC-MSC, embryonic stem cell–derived mesenchymal cells; GVHD, graft-vs.-host disease; HAPLN1, hyaluronan and proteoglycan link protein 1; IBD, inflammatory bowel disease; IL, interleukin; IFN-γ, interferon-γ; I/R, ischemia/reperfusion; LPS, lipopolysaccharide; miR, microRNA; Mφ, macrophages; MZB1, marginal zone B and B-1 cell-specific protein; OA, osteoarthritis; OPN, diabetic peripheral neuropathy; STAT, signal transducer and activator of transcription; T_H_, helper T cell; Treg, regulatory T cell; UC-MSC, umbilical cord–derived mesenchymal cells*.

### Immunomodulatory Effects of MSC-Derived EVs on T Cells

The effect of MSC-derived EVs on T cells has been described in several *in vitro* experiments. For example, Chen et al. co-cultured peripheral blood mononuclear cells with MSC-derived EVs and found that EVs induce the transformation of T_H_1 cells into T_H_2 cells, reduce the potential of T cells to differentiate into T_H_17 cells, and increase the content of Tregs (Chen et al., 2016). The regulatory effects of MSC-derived EVs on T cells have also been confirmed in various disease models. Cosenza et al. assessed the immunosuppressive effects of EVs on T cells in a delayed-type hypersensitivity model. The results showed that EVs from MSCs inhibited T-cell proliferation and induced Treg populations in a dose-dependent manner, thereby exerting an immunomodulatory effect on inflammatory arthritis (Cosenza et al., 2018). Zhang et al. further demonstrated that MSC-derived EVs induce the differentiation of naive T cells into Tregs via an APC-mediated pathway *in vitro* and *in vivo* (Zhang B. et al., 2018).

Owing to the plasticity of MSCs and the biological characteristics of EVs, EVs from modified MSCs have also been investigated in the field of inflammatory disease therapy. Riazifar et al. evaluated the role of EVs derived from MSCs stimulated by IFN-γ (IFN-γ-EVs) as a treatment in an experimental autoimmune encephalomyelitis mice model (Riazifar et al., 2019). They demonstrated that EVs decreased neuroinflammation and up-regulated the number of Tregs within the spinal region. Furthermore, RNA sequencing showed that IFN-γ-EVs contained anti-inflammatory RNAs and proteins, and inhibition of these RNAs could partially inhibit the potential of EVs to induce Tregs, suggesting potential for EVs as a cell-free therapy for immune-related diseases. Studies have also investigated molding EVs via lentivirus transfection of MSCs. Wei et al. developed an miR-181–overexpressing MSC-EV system that has strong therapeutic effects on myocardial I/R injury. The miRNA-181a mimic was able to interact with the c-Fos mRNA complex and induce Treg differentiation (Wei et al., 2019).

In conclusion, the immunoregulatory effects of MSC-derived EVs on T cells are manifested mainly in the immunosuppression of effector T cells and the induction of Tregs (Table 1).

### Immunomodulatory Effects of MSC-Derived EVs on B Cells

MSC-derived EVs also play an immunosuppressive role for B cells and can inhibit the terminal differentiation and maturation of plasma cells (Cosenza et al., 2018). In an OA model induced by collagenase, MSC-derived EVs effectively reduce the clinical symptoms of inflammation. However, treatment with IFN-γ did not affect the immunosuppressive function of EVs before isolation from MSCs (Cosenza et al., 2018).

A large number of studies have shown that EVs derived from MSCs exert effects through horizontal protein transfer, mRNA, and regulatory microRNA. Based on evidence that miRNA may be transferred to target cells, Khare et al. studied changes in activated B-cell mRNA after culture with bone marrow MSC (BMSC)-derived EVs and found that 186 genes were significantly differentially expressed after culture with EVs. The BMSC-derived EVs may inhibit the proliferation and decrease the chemotaxis of B cells by inhibiting the expression of chemokine receptor on B cells (Khare et al., 2018).

However, a recent study showed that the immunomodulatory effect of MSCs on B cells is mediated partially by soluble secreted factors rather than EVs. The authors found that MSC-derived EVs failed to induce naive B cells or reduce memory B cells, unliske the MSCs themselves. Furthermore, MSC-derived EVs induced CD24^+^CD38^+^ B cells to a similar extent compared with MSCs, but these B cells did not produce IL-10 and were therefore not considered true Bregs (Carreras-Planella et al., 2019b).

Do MSC-derived EVs have immunosuppressive effects upon B cells? The roles of EVs in MSC-mediated B-cell immunoregulation merit further investigation (Table 1).

## Sustained Delivery of EVS

One challenge in the application of sustained EV delivery systems is the difficulty of producing EVs in large quantities and with high purity (Yamashita et al., 2018). The typical yield of EVs isolated from 1 mL of medium may be <1 μg EV protein, whereas the effective dose of EVs in mouse models typically ranges from 10 to 100 μg of protein. Humans are expected to require larger therapeutic doses (Willis et al., 2017). The endocytic pathway of target cells is the main route by which EVs exert their biological effects (Mulcahy et al., 2014). However, studies have shown that, regardless of their source and whether they are administered through direct intravenous injection, subcutaneous injection or any other direct delivery routes, EVs will be quickly cleared from blood circulation and discharged from the body (Smyth et al., 2015; Charoenviriyakul et al., 2017). Therefore, prolonging the half-life of EVs to maximize their local therapeutic effects remains a major challenge.

To overcome this limitation, biomaterials may be utilized in EV encapsulation strategies to deliver EVs in a sustained-release manner. Hydrogels are three-dimensional cross-linked hydrophilic polymers with biological media that swell in water but do not dissolve (Narayanaswamy and Torchilin, 2019). Because hydrogels are biodegradable, biocompatible, and non-toxic, they have received great attention in various fields. Hydrogels can serve as biological drug delivery systems, cell encapsulation matrices, or scaffolds in regenerative medicine (Oliva et al., 2017; Mahdavinia et al., 2018). Hydrogel is considered to be an excellent carrier for EVs and may be capable of effectively delivering EVs to damaged tissues and prolonging their effects (Riau et al., 2019).

Liu et al. developed a photoinduced imine-crosslinking hydrogel glue to load-induced pluripotent stem cell–derived EVs for repairing knee articular cartilage defects, effectively prolonging the activity of stem cell–derived EVs promoting the repair of cartilage defects (Liu et al., 2017). Zhang et al. isolated EVs from human placenta-derived MSCs and combined EVs with chitosan hydrogel to form hydrogel-incorporated EVs (CS-EVs). They evaluated the effects of CS-EVs in endothelial cells using a murine hindlimb ischemia model and found that chitosan hydrogel strongly enhanced the retention of EVs at the target site. CS-EVs had advantageous functions compared with EVs alone, including enhancing angiogenesis, suppressing apoptosis of muscle cells, and attenuating fibrosis (Zhang K. et al., 2018). Henriques-Antunes et al. exploited a light triggerable hyaluronic acid hydrogel for the remote release control of EVs, which is more efficient in regulating EVs release according to the tissue healing dynamics in diabetic chronic wounds. Moreover, this hydrogel can provide antimicrobial activity by incorporating autolytic enzymes such as collagenases and antimicrobial peptides in order to avoid recurrent infection (Henriques-Antunes et al., 2019). Given that the mesh size of the ECM is usually smaller than the size of EVs, understanding how EVs are dispersed is critical to developing these cell-free therapies and stopping disease progression. Recently, Lenzini et al. revealed the diffusion and transport process of EVs in dense ECM by using engineered hydrogel. They confirmed that matrix stress relaxation is a key factor for EVs to overcome the confinement and that water infiltration also plays an essential role in EV deformation and navigation in ECM. These findings bring us closer to designing effective drug delivery systems (Lenzini et al., 2020).

## Perspective

The current viewpoint of OA is evolving from a simple mechanical joint injury toward a complex organ disease that involves biomechanics, inflammation, and the immune system. Macrophages, T cells, and B cells are the three main immune cells that control the inflammatory processes and immune response in OA. They form a complex network regulation system through coordination and antagonism. For example, T_H_2 cells can induce macrophage polarization via cytokines, yet the M2 polarization of macrophages might be involved in the induction of Tregs (Murray, 2017; Phan et al., 2017). Each of these processes overlaps and is also related to the local microenvironment, including trauma, inflammation, and cartilage remodeling. Fully understanding the complex inflammatory network system in OA will facilitate more effective and systematic treatment (Figure 1).

Currently, several studies have demonstrated the immunomodulatory effects of MSCs/EVs during OA, but there is still a lack of hard evidence of key factors and related mechanisms. Therefore, we also discussed the immunomodulatory effect of MSCs/EVs in some similar disease models based on chronic degenerative diseases with chronic inflammation hereinabove. We speculate that MSCs/EVs have similar immunomodulatory mechanisms in OA, and these roles need to be further investigated by future research in OA models.

As we have seen, MSCs have been widely used in regenerative therapy and immunotherapy owing to their anti-inflammatory and immunomodulatory effects (Prockop and Oh, 2012). The role of MSCs is to adjust the balance between inflammation and tissue reconstruction to provide the damaged tissue with a relatively stable environment, which is beneficial for tissue repair (Shi et al., 2018). However, direct MSC transplantation has several critical limitations to overcome: (1) the procurement of some stem cells, such as embryonic stem cells, is still controversial; (2) it is difficult to predict the lasting capacity for cell behavior and cell–cell interactions because of aging; (3) the survival rate associated with stem cell transplantation is low; (4) there is immune rejection in some patients; (5) the procedures are typically expensive and have a high risk of infection (Jiang and Xu, 2020). Moreover, as has been noted, MSCs are sensitive to certain microenvironment or inflammatory milieu and undergo specific behavioral modification. The plasticity of MSCs may have a negative impact on direct MSC transplantation. For example, MSCs can switch to MSC1, when induced by exposure to a low level of TLR2/4 ligands *in vivo*, and can then take effect in augmenting the inflammatory response (Tomchuck et al., 2008; Lei et al., 2011).

Therefore, more and more researchers have tended to test the secretory products of MSCs, including MSC-derived EVs in animal models of OA, rather than direct transplanting MSCs themselves. EVs derived from MSCs have been proven to reproduce some benefits of MSC-mediated immunosuppression (Mianehsaz et al., 2019). They can transfer miRNA or other bioactive substances such as cytokines, growth factors, and metabolites to target cells, thus playing the role of immune regulators. Being cell-free particles, EVs can avoid any chance of cell modification, and this guaranteed the stability of soluble bioactive factor types and abundance applied to the degenerative joint. Further knowledge regarding the mechanisms of action for MSCs and EVs in OA will enable individual assessments of the severity of the articular condition. Based on the plasticity of MSCs, it may be possible to obtain EVs with different characteristics prior to application to develop personalized treatments and increase therapeutic efficacy (Ti et al., 2015; Song et al., 2017; Jiang et al., 2018; Riazifar et al., 2019). However, EV-based therapy has some disadvantages: First, MSCs have numerous contact effects and numerous paracrine effects including EVs; therefore, EVs can reproduce the positive function of MSCs partially, but not all of them. Second, the major drawback of EVs is their rapid clearance, and sustained local delivery of EVs is therefore important (Smyth et al., 2015; Charoenviriyakul et al., 2017). It is also important to determine how to prepare EVs quickly, cheaply, and efficiently. Moreover, as described in *Immunomodulatory Effects of MSC-Derived EVs on B Cells*, a recent study different from previous studies reported no or low functional effects of EVs on B cells (Carreras-Planella et al., 2019b). This discrepancy may be due to a variety of factors, including MSC sources, isolation methods, culture conditions, and/or the use of repeated freeze-thawed EVs. Another possibility is that EVs play a small role in MSC-mediated B-cell immunoregulation, which need further investigation; if that is the case, the appropriate treatment (cell or cell-free) should be selected according to different targeted immune cells.

To sum up, EVs avoid some of the inherent risks of MSC therapies and hold great promise in a range of applications. However, there are still many uncertain aspects of the interactions between MSCs/MSC-derived EVs and immune cells involved in OA, and future studies should further explore and optimize the role of EVs in immunoregulation and enable the efficient application of EVs in clinical therapy.

## Author Contributions

XW and QY initiated the project and made suggestions and revised the article. XZ, YZ, XS, and YX searched the database, wrote, and finalized the manuscript. All authors reviewed and commented on the entire manuscript.

## Conflict of Interest

The authors declare that the research was conducted in the absence of any commercial or financial relationships that could be construed as a potential conflict of interest.
